# Evaluation of the notifiable diseases surveillance system in sanyati district, Zimbabwe, 2010-2011

**DOI:** 10.11604/pamj.2014.19.278.5202

**Published:** 2014-11-14

**Authors:** Brian Abel Maponga, Daniel Chirundu, Gerald Shambira, Notion Tafara Gombe, Mufuta Tshimanga, Donewell Bangure

**Affiliations:** 1 Department of Community Medicine, University of Zimbabwe, Zimbabwe; 2 Department of Health, Kadoma City Council, Zimbabwe

**Keywords:** Evaluation, notifiable disease surveillance, Sanyati, Zimbabwe

## Abstract

**Introduction:**

The Notifiable disease surveillance system (NDSS) was established in Zimbabwe through the Public Health Act. Between January and August 2011, 14 dog bites were treated at Kadoma Hospital. Eighty-six doses of anti-rabies vaccine were dispensed. One suspected rabies case was reported, without epidemiological investigations. The discrepancy may imply under reporting of Notifiable Diseases. The study was conducted to evaluate the NDSS in Sanyati district.

**Methods:**

A descriptive cross sectional study was conducted. Healthcare workers in selected health facilities in urban, rural, and private and public sector were interviewed using questionnaires. Checklists were used to assess resource availability and guide records review of notification forms. Epi Info^TM^ was used to generate frequencies, proportions and Chi Square tests at 5% level.

**Results:**

We recruited 69 participants, from 16 facilities. Twenty six percent recalled at least 9 Notifiable diseases, 72% correctly mentioned the T1 form for notification, 39% correctly mentioned the forms completed in triplicate and 20% knew it was a legal requirement to notify. Ninety six percent of respondents indicated willingness to participate, whilst 41% had ever received feedback. Three out of 16 health facilities had T1 forms.

**Conclusion:**

NDSS is useful, acceptable, simple, and sensitive. NDSS is threatened by lack of T1 forms, poor feedback and knowledge of health workers on NDSS. T1 forms and guidelines for completing the forms were distributed to all health facilities, public and private sector. On the job training of health workers through tutorials, supervision and feedback was conducted.

## Introduction

Public health surveillance is the ongoing, systematic collection, analysis, interpretation, and dissemination of data about a health-related event for use in public health action to reduce morbidity and mortality and to improve health [1]. A Notifiable disease is one required to be reported to local government health officials when diagnosed, because of infectiousness, severity, or frequency of occurrence [2].

The Notifiable Diseases Surveillance system (NDSS) supports case detection and public health interventions, estimates the impact of Notifiable diseases, portrays the natural history of Notifiable diseases, determines the distribution and spread of Notifiable diseases, generates hypotheses and stimulates research on Notifiable diseases, facilitates planning and evaluation of prevention and control measures and, facilitates the detection of outbreaks [1]. According to the revised Centre for Disease Control and Prevention (CDC) guidelines on evaluation of surveillance systems, the measurement of the performance of public health surveillance systems for outbreak detection is needed to establish the relative value of different approaches and to provide information needed to improve their efficacy for detection of outbreaks at the earliest stages.

According to the Zimbabwe Public Health Act (15:09), the following 19 diseases are Notifiable: Acute flaccid paralysis (AFP/polio), Anthrax, Brucellosis, Cholera, Diphtheria, Hepatitis (all forms), Human Influenza A caused by a new subtype (e.g. H1N1, H1N5), Meningococcal Meningitis, Noma, Plague, Rabies, SARS, Tuberculosis and Leprosy, Trypanosomiasis, Typhoid, Typhus, Viral Hemorrhagic fever (e.g. Ebola, Marburg, Crimean Congo), and Yellow fever [3].

The T1 form is used for notification of infectious diseases as described by the Public Health Act chapter 15:09, section 17 [3]. It is a case based system for prompt reporting of Notifiable diseases. Any health worker who comes in contact with a suspected or confirmed case of a Notifiable disease should immediately notify the District Medical Officer by telephone, radio or any other fast method available, within 24 hours of diagnosis. This is followed with a T1 form completed in triplicate [4]. If more than 5 cases of Notifiable cases of a disease occur in a specified time, a line list is maintained, which includes the first five cases [5].

The district compiles a summary of Notifiable diseases on the T2 form at the end of each month and summarizes all notifications for the month The T2 form completed by the district should reach the provincial office by the 10th of the following month [5]. The province summarizes all the districts T2 forms onto one Provincial T2 form, which is forwarded to the head office by the 24th of the month and a national summary is also produced [5]. Between 1^st^ January and 31^st^ August 2011, 14 cases of dog bites were referred from urban and rural clinics and treated at Kadoma General Hospital Outpatients Department (OPD). Eighty-six (86) doses of anti rabies vaccine were dispensed to victims of dog bites, translating to at least 22 potential cases of animal bites cases (assuming each case received the recommended 4 doses). However, a single case of suspected rabies was reported through the Notifiable Diseases Surveillance System to the district, and to the provincial office. There is no evidence of further epidemiological investigations and follow up of the animal bites cases to check if any developed rabies. This could be an indication of possible under reporting Notifiable diseases. This is a cause for concern to the district and provincial health management as this can result in untimely investigation and control of communicable diseases.

The study was conducted to evaluate the Notifiable Disease Surveillance System (NDSS) in Sanyati district, for the period January 2010 to June 2011. The study specifically set out to: describe the NDSS in Sanyati District; assess the health worker knowledge on the NDSS; assess the usefulness of the NDSS; assess the NDSS attributes; determine the cost of running the NDSS; and to come up with recommendations to improve the NDSS.

## Methods

A descriptive cross sectional study was conducted in selected health facilities in Sanyati district. The health facilities were in both the private and public sector, urban and rural settings in Sanyati District. Nurses, Environmental health personnel, and doctors in selected health facilities were recruited into the study. Selected Health managers from both the rural and urban public sector were selected as key informants. A minimum of 66 health workers and 30 T1 forms were to be recruited into the study.

The two hospitals, Sanyati Mission hospital, and Kadoma General Hospital and Rimuka clinic in urban Kadoma, were purposively selected, because of the large populations that the facilities serve. The clinics were selected by random sampling, selecting half of facilities in each stratum. The clinics were grouped into the following strata: Health facilities in rural Sanyati District (7 out of 14 selected), Kadoma city health facilities (2 out of 4 selected), and private health facilities (5 out of 10 private health facilities selected). All doctors, nurses and EHTs on duty, in the selected health facilities were interviewed. Random selection of T1 Notification forms covering the period January 2010 to June 2011 was used.

A pre tested interviewer questionnaire was administered to health workers, and managers. The questionnaires were used to collect information on the knowledge levels among the health workers and managers on the NDSS, assess usefulness, and attributes of the NDSS. Checklists were used to assess for resource availability, and for guiding the desk review of T1 Notification Forms. Outbreak reports, minutes of meetings, spot maps and graphs were checked to verify responses. Data were captured into Epi-Info^TM^ (CDC 2011). Epi-Info^TM^ was used to generate frequencies, proportions, and graphs and conduct Chi Square test for statistical significance. Permission was obtained from the local health authorities, and the Health Studies Office in Zimbabwe. Informed written consent was obtained from the study participants. Confidentiality was assured and maintained throughout the study.

## Results

Four key informants were interviewed. Sixty nine (69) health workers were interviewed. Fifty five (80%) of the respondents were from the private sector, whilst 14 (20%) were from private sector. Fifty six respondents were nurses, 9 were environmental health and four were doctors. The median duration in employment was five years in the public sector (Q_1_=2, Q_3_=11), and 11 years in private sector (Q_1_=7, Q_3_=13).

### Description of the T1 Surveillance System

In Sanyati district, the T1 surveillance system is paper based from the health centre/hospital level. Whenever a Notifiable disease is encountered at the health facilities notification is done using T1 forms are which completed in triplicate, but sometimes in duplicate and sent to the district office as soon as possible. The district office is notified immediately by phone or radio. The completed T1 forms are sent by health facility transport (motor cycle, or vehicle), according to 62% of the respondents, whilst a general hand or other health worker is sent using public transport with the forms (16%).

In the urban setting, no Notifiable disease had been reported in the period under review, but key informants indicated that the T1 forms are completed in triplicate, and a copy is sent to the City Health Director, and another copy forwarded to the District Office. An outbreak of cholera in early 2010 had not been notified using T1 forms, but line lists were completed. Feedback is through investigation of the reported cases by the district team, and through supervisory visits. There is no written feedback to the health facilities. Disease surveillance meetings are not done at the district level. Sixty Nine percent (69%) and 13% of respondents had received supervision within one month, and three months prior to the interview, respectively. Seventy three (73%) percent of the respondents mentioned that surveillance is discussed during visits. However, a review of visitors book shows that Vaccine Preventable Diseases (VPD's) and TB/HIV issues are mainly discussed during visits.

### Knowledge of Health Workers on NDSS

Table 1 summarizes the knowledge of health workers on the NDSS, in Sanyati district. The proportions of health workers trained on IDSR (public = 20%, private = 14%, p > 1.00) and NDSS new job induction (public = 33%, private = 43%, p = 0.54) was low. Knowledge on the correct form used (80%) was high in the public sector, compared to 43% in private sector (p = 0.02). Knowledge that notification should be done within 24 hours was high (79%) in the public (78) and private sector (86), p = 0.76. Health worker knowledge on the statutory requirement (public = 18%, private = 29%, p = 0.46), correct number of forms filled (public = 38%, private = 43%, p = 0.99) and knowledge of at least 9 Notifiable diseases (public = 29%, private = 14%, p = 0.33) was low.

**Table 1:** Knowledge of health workers on NDSS, Sanyati district, Zimbabwe, 2012

Attribute	Total n = 69 (%)	Public, n = 55 (%)	Private, n = 14 (%)	p Value
Were IDSR trained	13(19)	11(20)	2(14)	1.00
Were inducted in NDSS	24(35)	18(33)	6(43)	0.54
Knew notification was statutory requirement	14(20)	10(18)	4(29)	0.46
Mentioned ≥9 notifiable diseases	18(26)	16(29)	2(14)	0.33
Knew correct notification form is T1	50(73)	44(80)	6(43)	0.02
Knew T1 forms filled in triplicate	27(40)	21(38)	6(43)	0.99
Knew reporting is within 24 hours	54(79)	42(78)	12(86)	0.76

### System Attributes

#### Acceptability

High proportions of respondents were willing to participate in the NDSS (public = 100%, private = 79%, p = 0.007); and acknowledging responsibility of the diagnosing health worker to notify (public = 80%, private = 71%, p = 0.72). Overall, 7% mentioned it was the duty of the Environmental Health personnel to notify, whilst 12% did not know who was responsible for notifying. Reported feedback from higher level was low, (public = 47%, private = 50%, p = 0.91).

#### Data Quality

Sixty three (97%) of the respondents mentioned that the completed forms are checked by their supervisor for data quality before submission to the next level. Of the 12 completed T1 forms that were identified and reviewed, 10 were from one clinic (anthrax), and two were from the Mission Hospital (Cholera and Suspected Rabies). All 12 completed forms did not have the name of the province where they were to be sent, 11 did not have district name and 3 did not have health facility name. Three forms did not have a diagnosis, 3 forms did not have notifying officer's name. In terms of time, all 12 forms had their dates in logical sequence, and all 12 notifications being done on the day of diagnosis.

#### Flexibility

According to the Public Health Act, any disease can be notified as per the declaration of the Minister of Health and Child Care. Nationally, SARS, and Influenza Subtype A (H1N1 and H1N5) had been added. However, no new diseases had been notified using the T1 form in Sanyati district. A review of the T1 form shows that the T1 form has a section on diagnosis which is open, and type of diagnosis. Forty three percent of health workers knew that the T1 form can be used to notify new diseases (public = 44%, private = 36%, p = 0.82).

#### Simplicity

Key informants indicated that it was easy to orient staff in NDSS. Twenty nine health workers (42%) had completed the T1 form before. Of these, 10 said they could complete the form in less than 10 minutes; whilst 16 completed the form in more than 15 minutes. Nineteen respondents said completing the T1 form was easy and three, from public sector, mentioned that completing the form was difficult. The key informants indicated that it was easy to analyze the data on a T1 form as the form had few fields, depicting the person, place and time. Analysis was considered simple as the analysis is done case by case, and then aggregated into the T2 form, monthly.

### Stability of the Surveillance System

**Figure 1:**
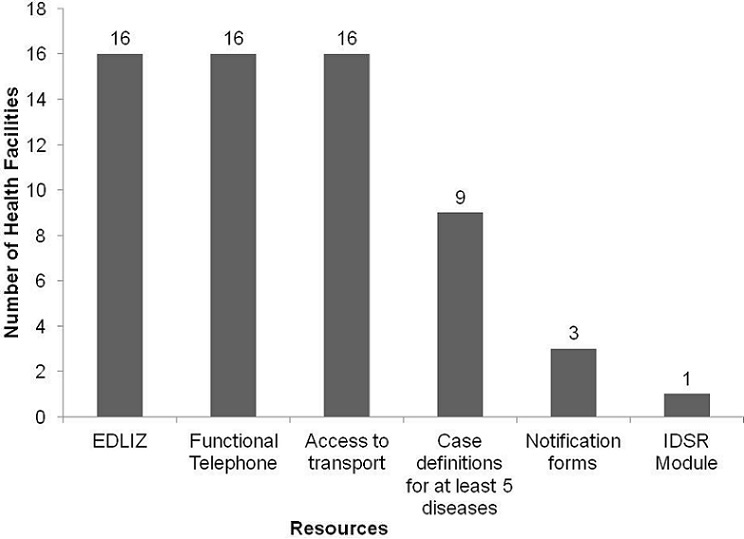
Availability of resources in NDSS in health facilities, Sanyati District, Zimbabwe, 2012

Figure 1 shows the availability of resources for NDSS, in the 16 health facilities. Only three health facilities had at least one T1 form, and none of the health facilities had at least 15 notification forms (enough to notify at least 5 cases). All 16 health facilities had the current Zimbabwe treatment guidelines (EDLIZ 5th Edition), whilst one clinic had a copy of the IDSR training guidelines. In terms of human resource availability, six of the 12 doctor's posts, 93% of nursing posts, nine out of the 14 environmental health posts, and all 3 health information posts were filled. There were no periods when there was no staff at all the health facilities, for the review period.

### Timeliness

Seventy-nine percent of respondents knew that notification should be done within 24 hours of diagnosing a Notifiable disease. All the 12 T1 forms reviewed at the district were notified on the date of diagnosis. However, there was no information as to when the district was notified by phone or when the forms arrived at the district.

**Sensitivity of Surveillance System** Four outbreaks of ND's were reported between January 2010, and June 2011. These were: one cholera outbreak in Kadoma city; one cholera outbreak rural Sanyati; one anthrax outbreak in Patchway and one suspected rabies outbreak in rural Sanyati. Three of the four outbreaks were reported using T1 forms. The cholera outbreaks in Kadoma City were not notified using T1 forms, but line lists were available.

### Representativeness of the surveillance system

Key informants indicated that all the health facilities in the private and public sector participate in NDSS. The proportion of the population who do not use the formal system in Sanyati district is not known, thus, could not determine the population covered by the NDSS.

### Usefulness

The health worker perception on usefulness of surveillance data and verification on usefulness were checked.

### Perceived Usefulness of NDSS

**Figure 2:**
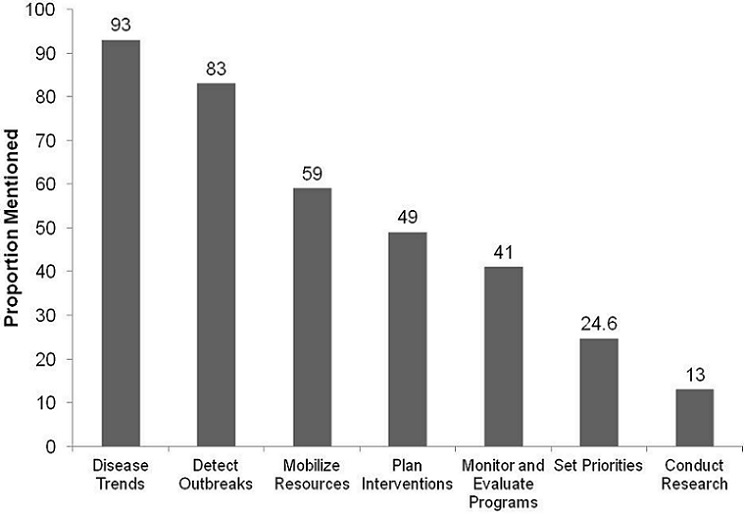
Health worker perception on usefulness of NDSS, Sanyati, Zimbabwe, 2012

Figure 2 shows the health worker perception of usefulness of the NDSS. High proportions of respondents perceived NDSS as useful: on use for monitoring disease trends (93%), and detection of outbreaks (83%). Low proportions of respondents perceived NDSS useful for: research (13%), and setting priorities (25%).

### Evident Usefulness of NDSS

Only three Notifiable diseases were reported during the period under review. Eleven of the 12 public health facilities had spot maps, which targeted mainly vaccine preventable diseases (VPDs). Use of NDSS data for planning and mobilizing resources was noted with the EPR cholera plan in Kadoma City; repair of radio communication system in rural Sanyati; vaccination of dogs and cattle in rural Sanyati, in response to rabies and anthrax outbreaks, after sharing information with respective stakeholders.

### The Cost of Operating the System

The average cost of notifying a single case was calculated, for the current paper based system. The cost was also calculated for an electronic system, if the NDSS adopt the mobile phone based method of data transmission, similar to the one being used for the weekly disease surveillance system (WDSS). The Table 2 summarizes the average cost of notifying a single case of Notifiable disease. The paper based system is more expensive, averaging US$18.15, compared to a situation where the mobile phone can be adapted to send T1 forms, US$1.55. For the paper based system, almost 90% of the cost is for transporting the T1 form to the district office.

**Table 2:** Cost of operating the NDSS, comparing paper based and electronic system, Sanyati district, Zimbabwe, 2012

Requirements	Paper Based System	Electronic-Mobile Phone
Reproducing T1 forms @$0.05 per form	0.15	0.05 (facility only)
Salaries: Assuming: Time to complete T1 form = 10 minutes, Time to send T1 form = 4 Hours, average salary of $300.00.)	7.00	0.50
Telephone bills (4 minutes)	1.00	1.00
Transport (Average $10.00)	10.00	-
Total	18.15	1.55

## Discussion

The study was conducted to assess the knowledge of health workers on the T1surveillance, assess attributes of the T1 surveillance system, assess usefulness of the system, assess the effect of training on T1 surveillance system and make recommendations on findings. In this study, knowledge of NDSS among health workers was low. Lack of knowledge can result in health workers having a low index of suspicion of cases of Notifiable diseases or failing to report Notifiable diseases, resulting in delayed investigations and control of outbreaks. In Nigeria, in an evaluation of the Notifiable Disease Surveillance System, Bawa S.B, et *al*, in 2003, found that 38% knew about the Notifiable disease surveillance [6]. In Sanyati, very few respondents were aware of the legal requirement to report ND's. Contrasting findings were obtained in Mutare by Kangwende R.A, et *al*, in 2003 of high awareness of legal requirements to notify [7]. Consistent findings obtained in the United States, where health workers were not aware of legal requirements to report, lacked knowledge of which diseases to report, and assumed that someone will notify [8].

In this study, the poor knowledge on the NDSS could be due to lack of training. This is consistent with several studies done in Zimbabwe which found that lack of training in surveillance threatened the performance of surveillance systems [9–11]. In Tsholotsho district, Sibanda C, et *al*, 2010, found that delay in notification of dog bites was as a result of lack of training in IDSR, in 73% percent of the respondents [9]. However, the studies did not assess the effect of training on performance of surveillance systems. In Nigeria, a quasci experimental study reported by Bawa S.B, et *al*, in 2004, demonstrated that training increased health worker awareness of the Notifiable surveillance system from 35.6% to 92%, completeness rose from 2.3% to 52%, and timeliness increased from 0% to 42.9%. [12] In Sanyati, the effect of training could not be assessed because there were very few available T1 records, which were from two health facilities.

Even though knowledge on the NDSS among health workers was low, it was highly acceptable, both in public (100%) and private sector (79%), p = 0.01. Feedback is known to increase participation in surveillance, and corrects mistakes, and thus improves knowledge and use of the surveillance system. This contrasts with results in Mutare district, 2003, where Kangwende R.A, et *al*, reported there was no feedback at all [7].

In terms of data quality, all twelve T1 forms were notified on date of diagnosis. Ninety seven percent of the respondents indicated that T1 forms are checked for quality before being sent to the next level. This is usually the duty of the sister in charge, yet only 3 out of 11 SIC's were oriented in NDSS. The completed T1 forms were of poor quality, as they lacked some important information such as diagnosis and name of health facility. This may pose challenges in conducting investigations, analysis of data and generating reports. Further, conclusions on the timeliness and completeness of T1 forms may not be generalizable in the district, as less than the targeted 30 T1 forms reviewed, from three health facilities.

In terms of stability, there is nearly adequate staffing at all health facilities, except for medical doctors where there was high vacancy rates, with all the 4 respondents being stationed in the urban setting. One rural hospital did not have a doctor at all, thus ascertaining some diagnoses might be compromised, with resultant missing or delayed diagnosis of cases. Only three health facilities had at least one T1 form. Non availability of T1 forms can discourage a health worker from notifying, further delaying outbreak investigations. In Shamva district, Chahuruva E, et *al*, in 2007, reported that there were no notification forms in health facilities. [13] Similar findings were observed in Nigeria by Bawa S, et *al*, 2002, who reported that 92% of health facilities did not have notification forms [6]. As a matter of strength, all public health facilities in Sanyati district had case based notification forms for vaccine preventable diseases(VPD's) such as Polio, Measles and Neonatal Tetanus. The district health team can ride on this strength to strengthen the notifiable disease surveillance system.

All health facilities visited had access to communication through telephone, mobile phone or radio. The health facilities can now communicate with the district as soon as a Notifiable disease is identified. The scenario contrasts with Tsholotsho in 2010, where 2 out of 8 health facilities had a functional telephone, as reported by Sibanda C, et *al* [9]. In future, the mobile phones may be used to conduct electronic notification, similar to the WDSS, as the bigger chunk of the cost of notifying a disease is through transporting the T1 forms to the next level. The mobile phones are not yet activated for internet, which could come in handy with regards to reference reading and electronic mailing.

A complex reporting system tends to discourage health workers from reporting. In Sanyati district, 10 out of 29 respondents indicated that completing a T1 form was either difficult or time consuming. In Tsholotsho, Sibanda C, et *al*, found that completing the T1 form was not time consuming and the form could be completed in less than 10 minutes [9]. This contrasts to findings in a Syrian study on private doctors, where Hsiu-Fen Tan, et *al*, 1997, reported that 65% of respondents were willing to report if the reporting procedure was simplified [14]. In South Africa, Abdool Karim S S, et *al*, 1996, found that 55% of the doctors considered notifying too laborious [6]. However, it is difficult to compare the two studies with the study we conducted as the majority of our respondents were nurses (81%), and very few were from private sector (20%).

When the data is collected, it has to be put to public health use. Knowledge on use of data was high for monitoring disease trends (93%), and lowest for use of surveillance data for conducting research. In Sanyati district, NDSS data had been used to vaccinate dogs and cattle, and for health education purposes, even though it was not documented. Similarly, in Tsholotsho district the data had been used for vaccination of dogs [9]. In Shamva, there was no evident use of the data [13].

## Conclusion

In Sanyati district, the NDSS is useful, acceptable, simple, sensitive and timeliness is good. The NDSS is threatened by lack of T1 forms, poor quality of the data on completed T1 forms, low feedback and lack of knowledge of health workers on the NDSS. Overall, the strength and weakness of NDSS were almost similar for the public and private health sector. The cost of disease notification is reasonable, but the time and cost of transporting the forms to the next level makes it unsustainable. T1 notification forms and guidelines for completing the forms were distributed to all health facilities, public and private sector. On the job training of health workers through tutorials, supervision and feedback was conducted. There is need for further research on the effect of training on surveillance systems. The Ministry of Health should consider developing an electronic based system, riding on availability of mobile phones being used for the WDSS.
